# Boosting adult neurogenesis to enhance sensory performance

**DOI:** 10.15252/embj.2019101589

**Published:** 2019-02-20

**Authors:** Marcela Lipovsek, Matthew S Grubb

**Affiliations:** ^1^ Centre for Developmental Neurobiology Institute of Psychiatry, Psychology and Neuroscience (IoPPN) King's College London London UK

**Keywords:** Neuroscience

## Abstract

Although most mammalian neurons are born prenatally, there are at least a couple of specialised niches in the adult rodent brain that continually generate new neurons throughout life. The potential functions conferred by this process of adult neurogenesis, however, remain obscure, despite a sizeable literature exploring the links between alterations in neurogenic capacity and changes in behavioural ability. A new study by Bragado Alonso *et al* (2019) offers a novel viewpoint on this question by describing a particularly clean way to manipulate adult neurogenesis. Specifically altering cell cycle dynamics in adult neural stem cells leads to an increase in new‐born neuron production without altering those extra cells’ morphological or functional properties. Moreover, mice with boosted adult neurogenesis are significantly better at discriminating highly similar sensory stimuli.

It may be controversial in humans (Kempermann *et al*, 2018), but in rodents, it is well established that the adult brain still makes new neurons. In the dentate gyrus of the hippocampus and in the olfactory bulb (OB), newly generated cells continue to integrate into existing circuitry throughout life. What's rather less established is what those new neurons are actually good for. The adult neurogenesis field is full of studies that have manipulated the process in some way and then tested rodents on particular tasks, in an attempt to directly link the production of new neurons to specific behavioural outcomes. But diverse approaches to manipulating adult neurogenesis and to assaying behaviour have—perhaps unsurprisingly—produced a broad spectrum of results. This is clearly exemplified by the many approaches that have aimed to impair the production or maturation of adult‐born cells, where different genetic (e.g. Sakamoto *et al*, 2014; Li *et al*, 2018), pharmacological (Breton‐Provencher *et al*, 2009) or irradiation‐based (e.g. Lazarini *et al*, 2009) strategies to suppress adult neurogenesis have been associated with varying effects (if any…) on rodent behaviour. The opposite strategy of experimentally *increasing* the number of adult‐born neurons has also produced diverse and somewhat contradictory results. For example, elevating new‐born cell numbers by preventing cell death is associated with better performance on pattern separation tasks when induced in the hippocampus (Sahay *et al*, 2011), but produces worse sensory discrimination when it occurs in the OB (Mouret *et al*, 2009). On the other hand, elevating OB new‐born numbers by long‐term sensory enrichment is associated with better performance on specific sensory tasks (Rochefort *et al*, 2002). Such discrepancies are undoubtedly due to multiple causes, but one crucial factor contributing to the diversity of findings is the problem of manipulation specificity. In other words, it is very hard to increase numbers of new‐born neurons without changing anything else about them.

In this issue of *The EMBO Journal*, Bragado Alonso *et al* (2019) take an important step in this direction. Taking advantage of a well‐characterised genetic means of controlling cell cycle length, they manipulate adult‐born neuronal number at source by speeding proliferation of neural stem cells (NSCs) in the subventricular zone (SVZ). Crucially, this boost to neuron production is transient and does not affect the resident SVZ stem cell population beyond the time of manipulation, resulting in extra OB interneurons in only a specific time‐locked cohort. Even more crucially, the genetic manipulation itself is only transiently present in SVZ NSCs and is absent from the resulting supernumerary OB cells. In this way, Bragado Alonso *et al* produce a time‐limited cohort of extra adult‐born neurons that are different *in number only*, and in all other respects are just like new‐born cells generated in normal conditions.

To achieve this, Bragado Alonso *et al* used a double‐inducible conditional mouse model in which the cell cycle regulators Cdk4 and cyclinD1 (together known simply as “4D”) were overexpressed specifically in adult NSCs with tight temporal control. By shortening the G1 phase of the cell cycle in SVZ NSCs over the course of 4 days in adult mice, proliferation in the niche was temporarily elevated. This resulted in a transient increase in the number of activated SVZ “B” and “C” cells—the dividing cell types producing neuroblasts that then migrate through the rostral migratory stream to integrate as new neurons in OB circuits. As a result, numbers of new‐born OB interneurons were increased 1 month after the cessation of NSC 4D overexpression.

But are these extra new neurons “normal”? Importantly, because cyclinD1 is degraded during the cell cycle and is crucial for Cdk4 function, the mature neurons produced from 4D‐overexpressing NSCs did not themselves have functional 4D overexpression. They did, however, retain a specific fluorescent marker protein, and this enabled Bragado Alonso *et al* to compare them with adult‐born OB cells produced from control NSCs. Studying a specific subtype of OB interneuron—superficial granule cells—they found that 4D+‐derived neurons were morphologically indistinguishable from controls in both dendritic branching and spine density. What's more, targeted electrophysiological recordings from both types of new‐born neuron found that their passive membrane properties, active spiking parameters and spontaneous synaptic inputs were all strikingly similar. Structurally and functionally, then, the extra new neurons produced by 4D overexpression in SVZ NSCs seem to be just like any other age‐matched new neuron integrating into OB networks.

This led to the obvious question: Do those extra, but normal, adult‐generated neurons make any difference to behavioural ability? At first, with straightforward tasks of olfactory discrimination, the answer to this was a resounding “no”—mice with transient 4D overexpression were completely normal on easy discrimination tasks involving very distinct odorants. Their behaviour was also no different on reasonably difficult tests that required them to differentiate between closely related odour stimuli. It was only when the sensory discrimination task was made as difficult as possible, requiring the differentiation of weak dilutions of closely related odorants, that having additional new‐born OB cells made a difference. On this task, mice with transient 4D overexpression in SVZ NSCs were slightly, but significantly better than their counterparts with control levels of adult neurogenesis. Boosting adult neuron production can therefore improve sensory performance when the going gets really tough.

By specifically elevating the production of adult‐born neurons without seemingly altering anything else about them, Bragado Alonso *et al* have found a particular behavioural circumstance in which having those extra cells is associated with improved performance. This is an important result for the field, demonstrating that a clean and temporary change in the rate of adult neurogenesis can result in altered sensory ability.

The paper also raises some important and interesting questions for future studies. First, given that the behavioural effect of 4D overexpression was observed on just a single test, does the enhanced performance hold over a wider range of (difficult) olfactory tasks involving diverse odours or odour mixtures? Second, precisely which types of OB interneurons underlie the improved behaviour? Different contributions to information processing in OB circuits have been ascribed to granule cells and glomerular layer interneurons (e.g. Fukunaga *et al*, 2014). Adult neurogenesis produces multiple subtypes of OB interneurons, all of which are boosted by 4D overexpression in SVZ NSCs. So, is the improved discrimination of dilute similar odorants specifically linked to having extra granule cells, or other cell types or combinations? Third, seeing as it is impossible to know for sure that two groups of cells are *not* different in any way, is there a chance that subtle but important phenotypic differences between 4D+‐generated and control cells could underlie the behavioural effect seen? In particular, given that only granule cells were compared in this study, could the properties of glomerular layer neurons be altered by 4D overexpression in SVZ NSCs? Finally, is the behavioural effect dependent upon having extra freshly generated cells, or could it be replicated by *any* increase in GABAergic signalling in mature OB circuits (Gschwend *et al*, 2015)?

We look forward to seeing progress on these questions in the near future. But perhaps more exciting are the implications of Bragado Alonso *et al*'s work that go beyond adult neurogenesis and olfaction. If 4D overexpression is truly phenotypically benign for the extra cells it produces, and if it can be used in tandem with neurogenesis‐inducing strategies to boost production of new neurons in areas of the adult brain that do not normally generate them (and that's a big “if”…), it has huge potential for improving regenerative capacity in the context of brain repair after illness or injury. In those cases, more new neurons—and, crucially, more new *normal* neurons—could make all the difference.
